# *Helicobacter pylori* Antibiotic Resistance in Russia: A Systematic Review and Meta-Analysis

**DOI:** 10.3390/antibiotics14050524

**Published:** 2025-05-19

**Authors:** Dmitrii N. Andreev, Alsu R. Khurmatullina, Igor V. Maev, Dmitry S. Bordin, Andrey V. Zaborovskiy, Sayar R. Abdulkhakov, Yury A. Kucheryavyy, Filipp S. Sokolov, Petr A. Beliy

**Affiliations:** 1 Department of Internal Disease Propaedeutics and Gastroenterology, Russian University of Medicine, 127473 Moscow, Russia; 2 Department of Propaedeutics of Internal Diseases, Gastroenterology and Hepatology, I.M. Sechenov First Moscow State Medical University (Sechenov University), 119048 Moscow, Russia; 3 Department of Pancreatic, Biliary and Upper Digestive Tract Disorders, A. S. Loginov Moscow Clinical Scientific Center, 111123 Moscow, Russia; 4 Department of General Medical Practice and Family Medicine, Tver State Medical University, 170100 Tver, Russia; 5 Department of Pharmacology, Russian University of Medicine, 127473 Moscow, Russia; 6 Department of Internal Diseases, Institute of Fundamental Medicine and Biology, Kazan (Volga Region) Federal University, 420008 Kazan, Russia; 7 Department of Primary Care and General Practice, Kazan State Medical University, 420012 Kazan, Russia; 8 Ilyinskaya Hospital, 143421 Krasnogorsk, Russia

**Keywords:** *Helicobacter pylori*, Russia, meta-analysis, antibiotic resistance, systematic review

## Abstract

**Objective**: This systematic review and meta-analysis aims to evaluate the temporal changes in *Helicobacter pylori* antibiotic resistance in Russia based on studies published over the past 15 years. **Materials and Methods**: We conducted a comprehensive literature search in MEDLINE/PubMed, EMBASE, the Russian Science Citation Index, and Google Scholar, following the PRISMA 2020 guidelines. Our meta-analysis was pre-registered in PROSPERO (CRD 420251025636). The inclusion criteria included original research, published in English or Russian in 2011–2024, involving antibiotic susceptibility testing in treatment-naive Russian adults using validated diagnostic methods. Two independent researchers selected studies and extracted data using standardized procedures, with methodological quality assessed via the Newcastle–Ottawa Scale. Pooled resistance rates were calculated using fixed/random-effects models in MedCalc 23.1.5 and Python 3.9.21, with meta-regression investigating temporal trends and subgroup analyses examining regional and methodological variations. **Results**: We identified 16 studies comprising 1206 *H. pylori* isolates. The pooled analysis of studies (2011–2025) revealed an overall clarithromycin resistance rate of 15.236%, with a significant temporal increase from 11.903% pre-2015 to 21.024% in 2020–2024 (*p* = 0.0049). Metronidazole showed consistently high pooled resistance (33.309%), while amoxicillin (1.828%), levofloxacin (19.014%), tetracycline (1.328%), and rifampicin (5.803%) maintained low resistance rates, and dual clarithromycin–metronidazole resistance was observed in 2.793% of isolates. Regional disparities were notable in the two largest cities of Russia, with 18.763% clarithromycin resistance in Moscow versus 28.540% in Saint-Petersburg. **Conclusions**: Russia surpasses the Maastricht VI Consensus resistance threshold for clarithromycin (15%), necessitating revision of empirical treatment strategies. The significant increase in clarithromycin resistance, potentially exacerbated by antibiotic use during the COVID-19 pandemic, underscores the urgent need for resistance-guided therapies and ongoing national surveillance programs to optimize *H. pylori* management.

## 1. Introduction

*Helicobacter pylori* (*H. pylori*) is an extremely common pathogen that invariably leads to chronic gastritis, a condition that can progress to severe complications such as peptic ulcer disease and gastric cancer [1,2,3]. In 1994, the World Health Organization classified *H. pylori* as a Group 1 carcinogen [4]. According to aggregated data, approximately 79% to 89% of non-cardia gastric cancer cases are attributed to *H. pylori* infection [5,6]. A recent meta-analysis estimated the global prevalence of *H. pylori* infection in adults between 2015 and 2022 to be 43.9% (95% CI: 42.3–45.5) [7]. In Russia, the prevalence of the *H. pylori* infection in 2019 was 35.6%, showing a declining trend but remaining relatively high compared to infection rates in many developed countries [8].

Currently, the rise in *H. pylori* antibiotic resistance is widely recognized as a global problem, significantly limiting the efficacy of many established eradication therapy (ET) regimens in different regions [9,10]. Indeed, the failure rate of some conventional protocols can reach 20–30% in the presence of resistance to one of the antibiotics used [11,12]. A meta-analysis of 120 studies demonstrated that clarithromycin resistance significantly reduces ET efficacy (OR: 0.682; 95% CI: 0.636–0.731), even more so than metronidazole resistance (OR: 0.843; 95% CI: 0.810–0.877) [13]. Additionally, the widespread use of antibiotics during the early stages of the COVID-19 pandemic may have contributed to increased resistance rates [14,15].

The Maastricht VI Consensus (2022) recommends a tailored approach to selecting ET regimens based on regional resistance levels, particularly to clarithromycin (threshold: 15%) and local data on the effectiveness of different treatment protocols [1]. A meta-analysis conducted in Russia in 2020 reported *H. pylori* resistance rates of 10.39% for clarithromycin (95% CI: 7.103–14.219), 33.95% for metronidazole (95% CI: 15.329–55.639), 1.35% for amoxicillin (95% CI: 0.281–3.202), 20.0% for levofloxacin (95% CI: 12.637–28.574), and 0.98% for tetracycline (95% CI: 0.353–2.163), as well as dual resistance to clarithromycin and metronidazole at 2.37% (95% CI: 1.136–4.345) [16]. However, recent data from the European Registry on *H. pylori* Management (HpEu-Reg) indicate the suboptimal efficacy of clarithromycin-based triple therapy in Russia, with success rates of less than 80% [17,18]. Furthermore, the latest studies (2024–2025) from Moscow reveal that *H. pylori* resistance to clarithromycin now exceeds the 15% threshold set by the Maastricht VI Consensus (2022), limiting the use of classical triple therapy in the Russian population [14,19]. Importantly, no newer meta-analyses have been conducted to summarize updated resistance data in Russia, including trends over time or period-specific assessments, leaving the current epidemiological landscape unclear.

This systematic review and meta-analysis aim is to evaluate the dynamics of *H. pylori* antibiotic resistance in Russia based on studies published over the past 15 years.

## 2. Materials and Methods

### 2.1. Search Strategy

The study protocol was pre-registered in the Prospective Register of Systematic Reviews (PROSPERO) database with the identification number CRD 420251025636. We conducted a comprehensive literature search following the Preferred Reporting Items for Systematic Reviews and Meta-Analyses (PRISMA) 2020 guidelines [20]. A completed PRISMA-P checklist can be accessed from the Supplementary Materials, Table S1.

Our search included multiple databases to ensure the thorough coverage of relevant studies, encompassing MEDLINE/PubMed, EMBASE, the Russian Science Citation Index (RSCI), and Google Scholar. We limited our search to studies published between 1 January 2011 and 20 March 2025 to capture recent evidence while maintaining a manageable scope.

For the English-language databases (MEDLINE/PubMed and EMBASE), we used a combination of MeSH terms and keywords: (“Helicobacter pylori”[MeSH] OR “H. pylori” OR “Helicobacter infection”) AND (“Drug Resistance, Microbial”[MeSH] OR “anti-bacterial agents”[MeSH] OR “antibiotic resistance” OR “antimicrobial resistance”) AND (“clarithromycin”[MeSH] OR “metronidazole”[MeSH] OR “amoxicillin”[MeSH] OR “tetracycline”[MeSH] OR “levofloxacin”[MeSH] OR “rifampicin”[MeSH]). For the RSCI, we employed equivalent Russian-language terms to identify relevant local studies. The Google Scholar searches incorporated English and Russian terms to capture additional publications. This time frame was selected to capture shifts in resistance patterns following key regional and global events, such as updates in clinical guidelines, COVID-19 pandemic, changes in antibiotic consumption, and the potential impact of antimicrobial control measures.

### 2.2. Eligibility Criteria and Quality Assessment

We established strict inclusion and exclusion criteria to ensure this study’s quality and relevance. For inclusion, studies needed to be original research articles published in peer-reviewed journals in either English or Russian. We focused on isolates received from the adult population (≥18 years) with *H. pylori* infection confirmed through standard diagnostic methods (^13^C urea breath tests, rapid urease tests, histology, or cultural in vitro studies). Studies had to report resistance rates for at least one of our target antibiotics and be conducted within Russia.

The exclusion criteria focused on studies involving special populations (e.g., immunocompromised or cancer patients) or small sample sizes (<20 isolates), non-original research such as reviews or case reports, and animal studies. We also excluded articles that used data from countries other than Russia. When encountering duplicate publications (either within the same database or across different ones), we carefully compared the studies and selected the most complete one for inclusion in our final analysis.

The methodological quality of all included publications was rigorously evaluated using the Newcastle–Ottawa Scale (NOS) for observational studies. Inter-rater reliability was evaluated using Cohen’s kappa (κ) statistic, with the following standard interpretation: κ values below 0.20 indicated poor agreement, and those of 0.21–0.40, 0.41–0.60, 0.61–0.80, and 0.81–1.00 reflected fair, moderate, good, and excellent agreement, respectively. Agreement, both on individual items and overall, was assessed using this standardized scale.

### 2.3. Data Extraction

For the screening process, two qualified healthcare specialists (I.V.M. and D.S.B.) independently evaluated all identified records using the AI-Powered Systematic Review Management Platform (Rayyan, https://www.rayyan.ai, accessed on 13 February 2025). The selection protocol followed a two-phase approach: (1) initial title/abstract screening against predefined inclusion/exclusion criteria, and (2) full-text assessment of potentially eligible studies to confirm eligibility. Discrepancies were resolved through consensus or consultation with a third reviewer.

Following the final selection of included studies, two additional researchers (Y.A.K. and F.S.S.) independently extracted data using a standardized form to minimize bias. This step focused on collecting relevant outcomes (e.g., resistance rates, sample characteristics) from the approved papers.

We implemented a quality control protocol, to ensure maximal data accuracy and completeness. This included direct communication with corresponding authors for methodological clarification when necessary and formal requests for sensitivity testing data in cases when published reports contained incomplete information.

### 2.4. Statistical Analysis

The primary analysis focused on calculating pooled resistance rates with 95% confidence intervals. We conducted thorough heterogeneity assessments using Cochran’s Q test and I^2^ statistics to understand variations between studies.

We performed several subgroup analyses to explore potential sources of variation, including comparisons regarding study quality (using NOS scores), diagnostic methods, the cities in which the studies were conducted, and temporal trends. For the temporal analysis, we additionally employed meta-regression techniques using Python 3.9.21 (Amsterdam, The Netherlands). The meta-regression was conducted using a Generalized Linear Model (GLM) with a binomial family and inverse-variance weighting (1/SE^2^). The model included study year as a continuous predictor. Standard errors and 95% confidence intervals were calculated for each data point.

For the chronological analysis of resistance dynamics, the studies were divided into three periods based on isolate collection: Period 1 (pre-2015), Period 2 (2015–2019), and Period 3 (2020–2024). To assess potential publication bias, we utilized funnel plots and statistical tests (Begg–Mazumdar and Egger’s tests).

All statistical analyses were conducted using specialized software. We used MedCalc 23.1.5 (Ostend, Belgium) for the primary meta-analysis and Python 3.9.21 to construct forest-plots on Microsoft Windows 11 (Microsoft Corporation, Redmond, WA, USA).

## 3. Results

### 3.1. Search Results

**Figure 1:**
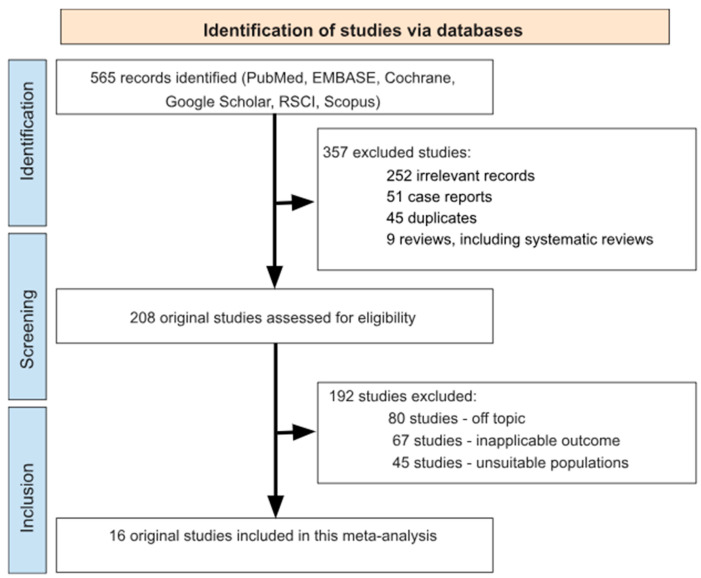
A flow chart detailing the study selection strategy.

Our systematic search of the electronic databases initially identified 565 potentially relevant scientific articles. After title/abstract screening, we excluded 357 publications for the following reasons: 252 were off topic, 51 were case reports, 45 were duplicate publications, and 9 were review articles (Figure 1).

The remaining 208 studies underwent rigorous full-text evaluation against our predefined inclusion criteria. This detailed assessment led to the exclusion of an additional 192 articles that failed to meet our methodological standards (80 had inapplicable outcomes, 67 involved insufficient patient populations, and 45 included unsuitable populations). Ultimately, only 16 original studies satisfied all eligibility requirements and were included in our final meta-analysis (Figure 1, Table 1) [14,19,21,22,23,24,25,26,27,28,29,30,31,32,33,34].

### 3.2. Characteristics of Included Studies

The final analysis included 16 studies conducted across various Russian regions and published between 2011 and 2025 [14,19,21,22,23,24,25,26,27,28,29,30,31,32,33,34], encompassing 1206 *H. pylori* isolates. The most significant number of studies came from Moscow (four studies [14,19,22,31]), Saint Petersburg (three studies [25,27,33]), Smolensk (three studies [21,28,30]), and Kazan (three studies [24,34]). Single studies from Novosibirsk [23], Vladivostok [26], Kursk [29], Chita [32], and Yaroslavl [31] were also included, ensuring reasonable geographical representation. For antibiotic susceptibility testing, seven studies used molecular genetic testing [19,22,23,24,29,32,34], five employed the serial dilution method [21,26,27,28,30], and four applied the disk diffusion method [14,25,31,33].

### 3.3. Study Quality Evaluation, Reliability Analysis and Publication Bias

Three studies demonstrated high methodological rigor (NOS 8–9), while ten exhibited moderate limitations (NOS 6–7), and three studies showed significant deficiencies (NOS ≤ 5). The primary factor compromising study quality was the limited representativeness of populations, with 37.5% of publications (*n* = 6) employing small sample sizes (<50 isolates). All included studies comprehensively documented the total number of analyzed isolates and corresponding resistance rates. We quantitatively assessed inter-rater reliability using Cohen’s kappa (κ) statistic; our analysis yielded excellent reliability metrics, with κ = 0.78 (substantial agreement) for study selection and κ = 0.92 (near-perfect agreement) for data extraction. We assessed publication bias for each antibiotic type; results can be found in Supplementary Material, File S2. Neither test provided statistically significant evidence of publication bias (*p* > 0.05 for both Egger’s and Begg’s tests).

### 3.4. Pooled Resistance Rates

**Figure 2:**
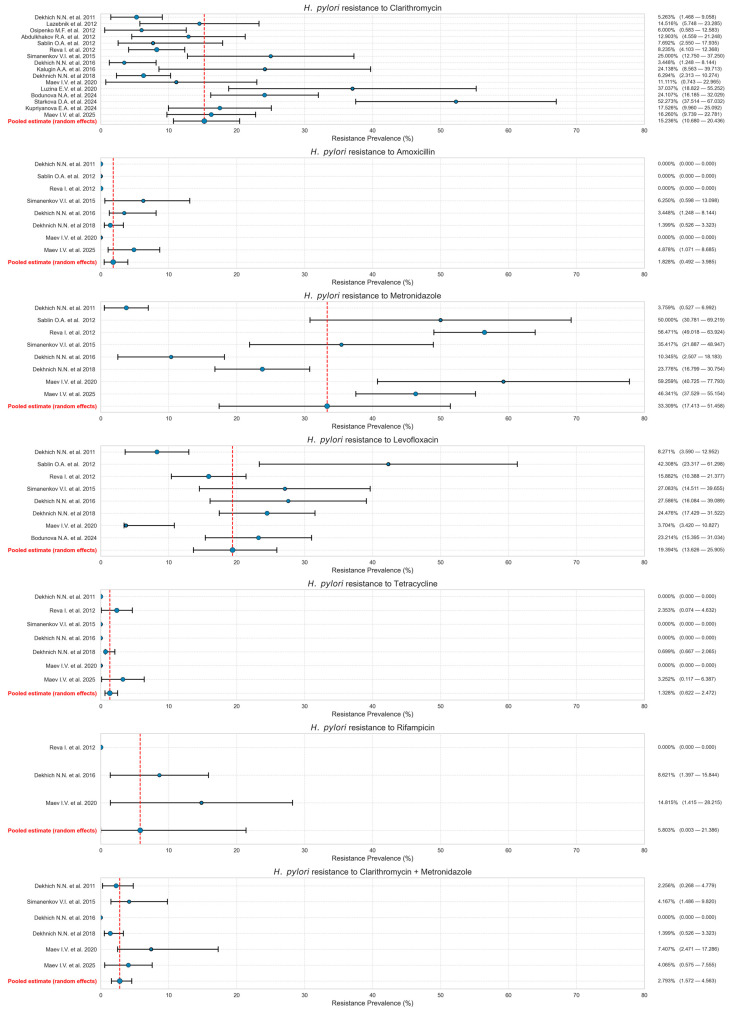
Overall prevalence of *H. pylori* resistance to analyzed antibiotics [14,19,21,22,23,24,25,26,27,28,29,30,31,32,33,34].

The pooled analysis of studies (2011–2025) revealed the following *H. pylori* resistance rates: 15.236% to clarithromycin (95% CI: 10.680–20.436;), 1.828% to amoxicillin (95% CI: 0.492–3.985), 33.309% to metronidazole (95% CI: 17.413–51.458), 19.394% to levofloxacin (95% CI: 13.626–25.905), 1.328% to tetracycline (95% CI: 0.622–2.472), and 5.803% to rifampicin (95% CI: 0.003–21.386). Dual resistance to clarithromycin and metronidazole was observed in 2.793% of isolates (95% CI: 1.572–4.563). These results are presented in Figure 2. A random-effects model was used to analyze resistance to clarithromycin, amoxicillin, metronidazole, levofloxacin, and rifampicin due to significant heterogeneity (I^2^ > 50%). In comparison, while fixed-effects models were applied for tetracycline and dual clarithromycin–metronidazole resistance (I^2^ < 50%). The analysis of studies published between 2015 and 2025 showed increased rates of resistance compared to resistance rates before 2015 to clarithromycin (19.014%; 95% CI: 12.007–27.191), amoxicillin (2.977%; 95% CI: 1.472–5.318), and metronidazole (35.760%; 95% CI: 20.087–53.184), with levofloxacin resistance remaining stable (18.277%; 95% CI: 12.213–25.251). Dual resistance to clarithromycin and metronidazole was found in 2.653% of isolates (95% CI: 1.247–4.905).

Additionally, we conducted a separate analysis to evaluate the resistance rate in the last 10-year period. The pooled prevalence of resistance to various antibiotics in *H. pylori* isolates collected between 2015 and 2024 revealed the following patterns: the rate of clarithromycin resistance was 19.014% (95% CI: 12.007–27.191), the rate of resistance to amoxicillin was 2.977% (95% CI: 1.472–5.318), and that to metronidazole was the highest at 35.760% (95% CI: 20.087–53.184). Resistance to levofloxacin was observed in 18.277% of cases (95% CI: 12.213–25.251), while the bacterium maintained relatively low resistance to tetracycline at 1.565% (95% CI: 0.550–3.468). Dual resistance to clarithromycin and metronidazole was found in 2.653% of isolates (95% CI: 1.247–4.905).

### 3.5. Chronological Dynamics of Antibiotic Resistance in H. pylori

For the chronological analysis of resistance dynamics, the studies were divided into three periods based on isolate collection: Period 1 (pre-2015) [21,22,23,24,25,26,27,33], Period 2 (2015–2019) [14,19,28,29,30,31,32,33,34], and Period 3 (2020–2024) [14,19,34]. One study [33] was excluded from the final trend analysis due to limited isolates in Period 3 (*n* = 5). This periodization enabled evaluating the evolution of resistance, including post-COVID-19 changes.

**Figure 3:**
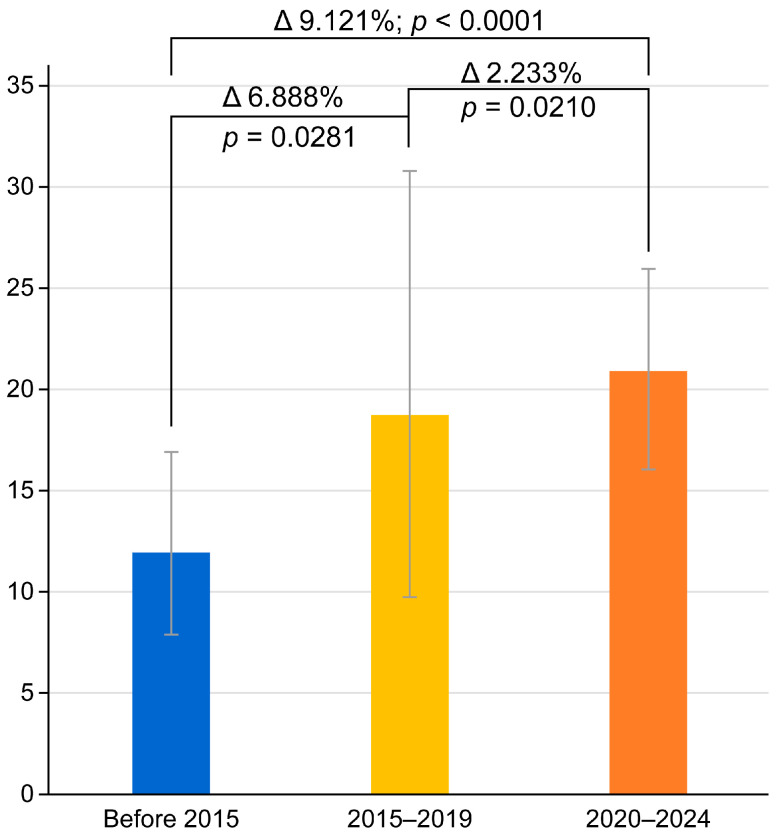
Pooled prevalence of *H. pylori* resistance to clarithromycin across three analyzed periods.

**Figure 4:**
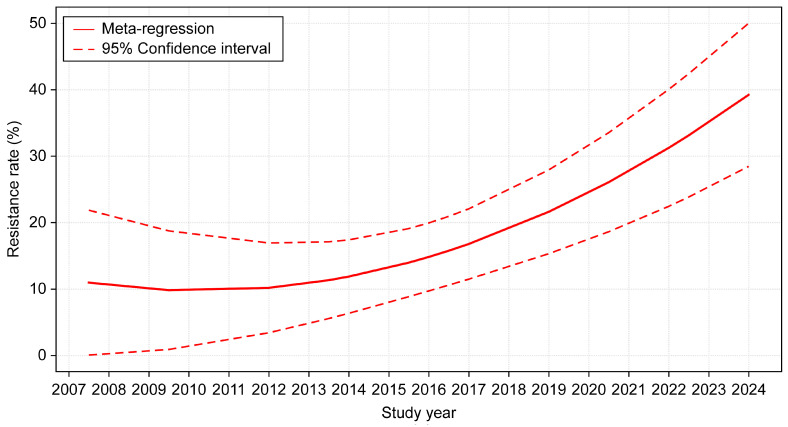
Meta-regression analysis of *H. pylori* clarithromycin resistance trend with 95% confidence interval.

The study revealed stable patterns of *H. pylori* resistance to amoxicillin, metronidazole, and tetracycline in Russia (Table 2). However, a concerning upward trend was observed for clarithromycin resistance, increasing from 11.903% (95% CI: 7.602–17.013) in the pre-2015 period to 21.024% (95% CI: 16.086–26.680) during 2020–2024 (Figure 3). The meta-regression analysis confirmed this temporal increase as statistically significant (*p* = 0.0049), with an annual regression rate of 1.810% (95% CI: 1.193–2.427) (Figure 4). In contrast, the apparent decline in levofloxacin resistance across the study periods did not reach statistical significance (*p* > 0.05 for all inter-period comparisons, Fisher’s exact test). These findings highlight the evolving challenge of clarithromycin resistance in *H. pylori* treatment regimens within the Russian population.

### 3.6. Subanalyses

We also subanalyzed *H. pylori* resistance rates across different antimicrobial susceptibility testing methods. Overall, the resistance patterns were generally consistent between methodologies, with two notable trends: there were lower detection rates of clarithromycin resistance when the serial dilution method was used, and a higher metronidazole resistance frequency was observed using disk diffusion testing (Table 3). These methodological differences highlight the importance of testing approaches when interpreting resistance data. This may be due to known limitations of this method for anaerobic organisms like *H. pylori*. Disk diffusion can overestimate resistance because metronidazole’s diffusion in agar is less reliable, and standard interpretive criteria for *H. pylori* are not well established. These methodological differences likely contribute to the variation in resistance rates observed across studies.

Additionally, we performed a separate subanalysis of the overall frequency of *H. pylori* resistance to various anti-bacterial agents across different cities in Russia, presented in Table 4. Regional disparities were notable in the two largest cities of Russia, with 18.763% clarithromycin resistance in Moscow versus 28.540% in Saint-Petersburg.

### 3.7. Sensitivity Analysis

We conducted additional sensitivity analysis to assess publication bias. In the first group (NOS < 7), the clarithromycin resistance rate was 12.434% (9.331–17.326), I^2^ = 68.09% (17.65–87.64). In the second group (NOS > 7), the clarithromycin resistance rate was 16.778 (14.791–18.922), I^2^ = 38.81% (0–80.96).

## 4. Discussion

*H. pylori* is one of the most common human pathogens, causing various gastroduodenal diseases, including gastric cancer [1,6]. Eradication therapy (ET) using PPI–antibiotic combinations can resolve gastric inflammation and prevent precancerous conditions such as atrophic gastritis and intestinal metaplasia [1,6,35,36]. However, antibiotic resistance significantly reduces the effectiveness of conventional ET protocols [13,37]. The development of *H. pylori* resistance primarily involves point mutations in specific genes that alter drug mechanisms, including those of clarithromycin (23S rRNA V domain), metronidazole (*rdxA*, *frxA*), amoxicillin (*pbp1A*), tetracycline (16S rRNA), and levofloxacin (*gyrA*) [19,38]. Cross-resistance poses a significant risk during antibiotic therapy, exacerbated by the COVID-19 pandemic’s disruption of global and Russian antibiotic consumption patterns [39,40,41]. In Russia, this period was characterized by a substantial rise in macrolide use, which may have accelerated resistance development in *H. pylori* to these critical antibiotic classes. Notably, azithromycin consumption exhibited dramatic shifts between 2018 and 2020: starting at 1.5 defined daily doses per 1000 inhabitants per day (DID) in 2018, it surged to 3.3 DID in 2020 —a 120% increase— primarily driven by outpatient care setting [41].

This systematic review and meta-analysis aimed to evaluate the dynamics of *H. pylori* antibiotic resistance in Russia. The analysis incorporated 16 studies comprising 1206 *H. pylori* isolates. The pooled prevalences of *H. pylori* resistance were as follows: 15.236% to clarithromycin, 1.828% to amoxicillin, 33.309% to metronidazole, 19.394% to levofloxacin, and 1.328% to tetracycline. The analysis of studies from the most recent 10-year period (2015–2025) revealed increasing resistance trends for nearly all antibiotics, with an increase of 19.014% for clarithromycin, 2.977% for amoxicillin, 35.760% for metronidazole, 18.277% for levofloxacin, and 1.565% for tetracycline. These findings align with recent data from the Hp-EuReg study that showed European resistance rates of 22% for clarithromycin, 27% for metronidazole, and 18% for fluoroquinolones [37]. Similarly, a recent global meta-analysis of 10-year antibiotic resistance data reported European resistance rates of 21.3% for clarithromycin and 29.9% for metronidazole [42].

The most significant finding of our study was the marked increase in *H. pylori* resistance to clarithromycin, which rose from 11.903% in isolates collected before 2015 to 21.024% in those collected during 2020–2024. This trend was statistically confirmed using Fisher’s exact test (*p* < 0.0001) and meta-regression analysis (*p* = 0. 0049). These findings are consistent with recent molecular genetic studies from Moscow and Kazan; Bodunova et al. (Moscow, 2024) identified clarithromycin-resistance mutations in 24.000% of samples [19], while Kuprivanova et al. (Kazan, 2024) found resistant strains in 17.530% of cases [34]. Notably, pre-2020 meta-analyses reported Russian clarithromycin resistance rates not exceeding 15% [16,43]. This concerning increase likely reflects Russia’s extensive macrolide use for respiratory infections (particularly during COVID-19) and prevalent self-medication practices [44].

These findings underscore the necessity for a tailored approach to selecting eradication therapy (ET) regimens based on regional antibiotic resistance patterns, as recommended by the Maastricht VI Consensus (2022) [1]. This aligns with current Russian clinical guidelines (2022), which specify that all *H. pylori*-positive patients undergoing ET should receive one of the following first-line 14-day regimens to ensure high eradication rates: (1) standard triple therapy with bismuth potassium citrate (PPI + clarithromycin + amoxicillin + bismuth); (2) classic quadruple therapy with bismuth potassium citrate (PPI + tetracycline + metronidazole + bismuth); or (3) non-bismuth quadruple therapy (PPI + clarithromycin + amoxicillin + metronidazole) [45]. These treatment protocols demonstrate high efficacy in eradicating *H. pylori*, as confirmed by multiple meta-analyses [46,47] and recent data from the European Registry on *H. pylori* Management (Hp-EuReg) in Russia [18].

The rising global threat of *H. pylori* antimicrobial resistance has reached a critical point. In 2018, the World Health Organization designated clarithromycin-resistant *H. pylori* as a high-priority pathogen requiring urgent intervention [48]. Our findings demonstrate that Russia has crossed the Maastricht VI Consensus threshold, with clarithromycin resistance rates reaching 21.024% (95% CI: 16.086–26.680) in recent years. This concerning trend mirrors patterns observed in other parts of the world and directly impacts clinical practice.

This meta-analysis has several limitations that should be acknowledged. First, the total number of analyzed isolates (*n* = 1206) remains relatively small compared to similar meta-analyses conducted in Europe, North America, and Asia [49,50,51], potentially affecting the generalizability of the findings. Second, the restriction of included studies to those published in Russian or English may introduce language bias, excluding relevant data from regional publications in other languages. Additionally, uneven geographic representation—with some federal districts of Russia contributing disproportionately fewer isolates—could lead to regional underrepresentation in the pooled resistance rates. Third, significant heterogeneity exists among the included studies, primarily due to variations in methodologies for assessing *H. pylori* susceptibility (e.g., differences in breakpoints, testing protocols, or sample collection periods), which may confound direct comparisons and pooled estimates. These methodological discrepancies underscore the need for standardized protocols in future surveillance efforts to improve data harmonization. Despite these limitations, this study represents the first comprehensive effort to systematically evaluate and quantify *H. pylori* antibiotic resistance dynamics across Russia. Given the rapidly evolving nature of resistance, these findings highlight the critical need for ongoing, large-scale multicenter studies that incorporate substantially greater numbers of isolates from all federal districts to ensure representative and up-to-date resistance data.

## 5. Conclusions

This systematic review and meta-analysis reveal a progressive increase in *H. pylori* clarithromycin resistance across Russia, which may reduce the effectiveness of current eradication therapies. These findings highlight the need for ongoing resistance monitoring, the regular evaluation of treatment efficacy, and the implementation of optimized therapeutic approaches—including resistance-guided regimens and alternative first-line protocols—to ensure successful *H. pylori* management in light of evolving antimicrobial resistance patterns.

## Figures and Tables

**Table 1:** Characteristics of selected studies.

Authorship and Year	City	Timeframe of Isolate Collection	Methodology	Number of Isolates	CLA-R	AMO-R	MET-R	LEV-R	TET-R	RIF-R	CLA-MET-R	NOS
Dekhich N.N. et al., 2011 [21]	Smolensk	2009–2010	Serial Dilution Method	133	7	0	5	11	0	n/a	3	7
Lazebnik et al., 2012 [22]	Moscow	Before 2012	Molecular Genetic Testing	62	9	n/a	n/a	n/a	n/a	n/a	n/a	6
Osipenko M.F. et al., 2012 [23]	Novosibirsk	Before 2012	Molecular Genetic Testing	50	3	n/a	n/a	n/a	n/a	n/a	n/a	6
Abdulkhakov R.A. et al., 2012 [24]	Kazan	Before 2012	Molecular Genetic Testing	62	8	n/a	n/a	n/a	n/a	n/a	n/a	7
Sablin O.A. et al., 2012 [25]	Saint-Petersburg	2012	Disk Diffusion Method	26	2	0	18	11	n/a	n/a	n/a	8
Reva I. et al., 2012 [26]	Vladivostok	2004–2009	Serial Dilution Method	170	13	0	96	27	4	0	n/a	7
Simanenkov V.I. et al., 2015 [27]	Saint-Petersburg	2013–2014	Serial Dilution Method	48	12	3	17	13	0	n/a	2	7
Dekhich N.N. et al., 2016 [28]	Smolensk	2015–2016	Serial Dilution Method	58	2	2	6	16	0	5	0	5
Kalugin A.A. et al., 2016 [29]	Kursk	2016	Molecular Genetic Testing	29	7	n/a	n/a	n/a	n/a	n/a	n/a	4
Dekhnich N. et al., 2018 [30]	Smolensk	2015–2017	Serial Dilution Method	143	9	2	34	35	1	n/a	2	8
Maev I.V. et al., 2020 [31]	Moscow, Yaroslavl	2015–2018	Disk Diffusion Method	27	3	0	16	1	0	4	2	7
Luzina E.V. et al., 2020 [32]	Chita	2019	Molecular Genetic Testing	27	10	n/a	n/a	n/a	n/a	n/a	n/a	5
Bodunova N et al., 2024 [19]	Moscow	2022–2023	Molecular Genetic Testing	112	27	n/a	n/a	26	n/a	n/a	n/a	7
Starkova D et al., 2024 [33]	Saint-Petersburg	2014–2022	Disk Diffusion Method	44	23	n/a	n/a	n/a	n/a	n/a	n/a	6
Kupriyanova E.A. et al., 2024 [34]	Kazan	2019–2021	Molecular Genetic Testing	97	17	n/a	n/a	12	n/a	n/a	n/a	7
Maev I.V. et al., 2025 [14]	Moscow	2015–2024	Disk Diffusion Method	123	20	6	57	n/a	4	n/a	5	8

CLA-R—Clarithromycin resistance; AMO-R—Amoxicillin resistance; MET-R—Metronidazole resistance; LEV-R—Levofloxacin resistance; TET-R—Tetracycline resistance; RIF-R—Rifampicin resistance; CLA-MET-R—Dual clarithromycin and metronidazole resistance; NOS—Newcastle–Ottawa Scale.

**Table 2:** Dynamics of resistance (%) to key antibiotics by isolate collection period.

Antibiotic	Before 2015	2015–2019	2020–2024
Clarithromycin	11.903% (95% CI: 7.602–17.013)	18.791% (95% CI: 9.258–30.727)	21.024% (95% CI: 16.086–26.680)
Metronidazole	33.542% (95% CI: 6.645–68.477)	33.412% (95% CI: 15.414–54.372)	1 study, n/a for meta-analysis
Amoxicillin	0.978% (95% CI: 0.000968–3.753)	1.955% (95% CI: 0.645–4.475)	1 study, n/a for meta-analysis
Levofloxacin	20.831% (95% CI: 10.481–33.591)	18.322% (95% CI: 10.144–28.262)	17.122% (95% CI: 6.010–32.399)
Tetracycline	0.958% (95% CI: 0.0224–3.244)	0.831% (95% CI: 0.113–2.847)	1 study, n/a for meta-analysis
Clarithromycin + Metronidazole	3.180% (95% CI: 1.153–6.867)	3.088% (95% CI: 0.530–7.660)	1 study, n/a for meta-analysis

**Table 3:** Antibiotic resistance rates (%) by testing methodology.

Antibiotic	Molecular Genetic Testing	Serial Dilution Method	Disk Diffusion Method
Clarithromycin	18.494% (95% CI: 13.138–24.532)	8.545% (95% CI: 4.491–13.742)	21.122% (95% CI: 6.817–40.608)
Metronidazole	n/a	23.752% (95% CI: 6.518–47.458)	48.889% (95% CI: 41.604–56.198)
Amoxicillin	n/a	1.526% (95% CI: 0.204–4.047)	2.955% (95% CI: 0.573–7.102)
Levofloxacin	17.876% (95% CI: 8.676–29.491)	19.740% (95% CI: 12.539–28.111)	20.537% (95% CI: 0.052–65.345)
Tetracycline	n/a	0.951% (95% CI: 0.260–2.069)	2.985% (95% CI: 0.884–6.272)
Clarithromycin + Metronidazole	n/a	2.060% (95% CI: 0.891–4.026)	5.134% (95% CI: 2.206–9.193)

**Table 4:** Pooled prevalence of *H. pylori* antibiotic resistance across major Russian cities.

Antibiotic	Moscow	Saint-Petersburg	Kazan
Clarithromycin	18.763% (95% CI: 14.014–24.028)	28.540% (95% CI: 11.376–49.776)	16.279% (95% CI: 10.953–22.882)
Metronidazole	46.398% (95% CI: 37.436–55.534)	27.612% (95% CI: 7.795–53.813)	n/a
Amoxicillin	3.791% (95% CI: 0.111–12.314)	3.769% (95% CI: 0.075–12.630)	n/a
Tetracycline	3.530% (95% CI: 1.052–8.448)	n/a	n/a
Levofloxacin	13.736% (95% CI: 1.239–36.503)	n/a	13.015% (95% CI: 7.093–21.271)
Dual Therapy (Clarithromycin + Metronidazole)	4.593% (95% CI: 1.657–9.881)	n/a	n/a

## Data Availability

No new data were created or analyzed in this study. Data sharing is not applicable to this article.

## Supplementary Materials

The following supporting information can be downloaded at: https://www.mdpi.com/article/10.3390/antibiotics14050524/s1, File S1. Completed PRISMA-P checklist; File S2. Risk of Bias Assessment.
