# Supplementary material for: Helicobacter pylori Antibiotic Resistance in Russia: A Systematic Review and Meta-Analysis
Source: Antibiotics (Basel). 2025 May 19;14(5):524. doi: 10.3390/antibiotics14050524 (PMC12108198; doi:10.3390/antibiotics14050524)

Clarithromycin


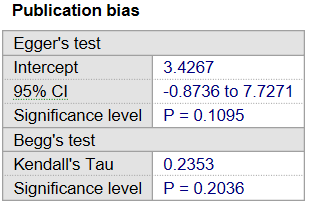


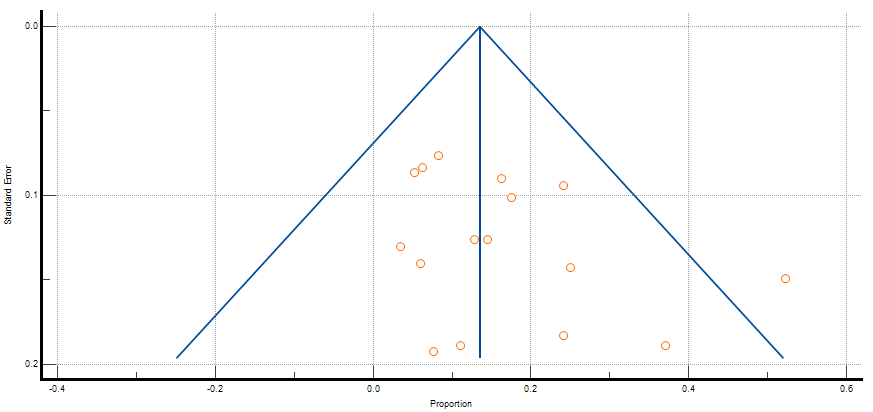


Amoxicillin


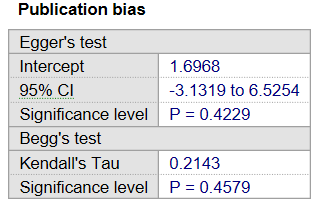


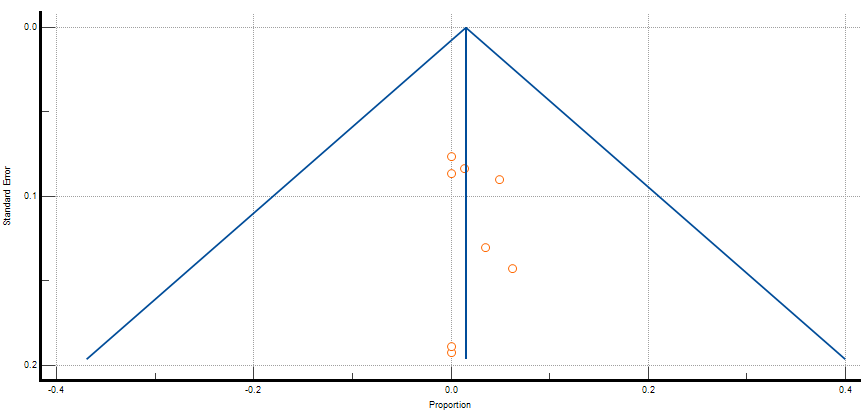


Metronidazole


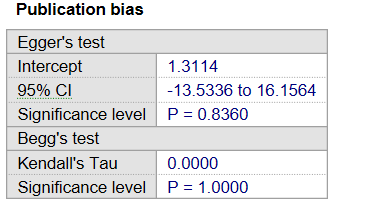


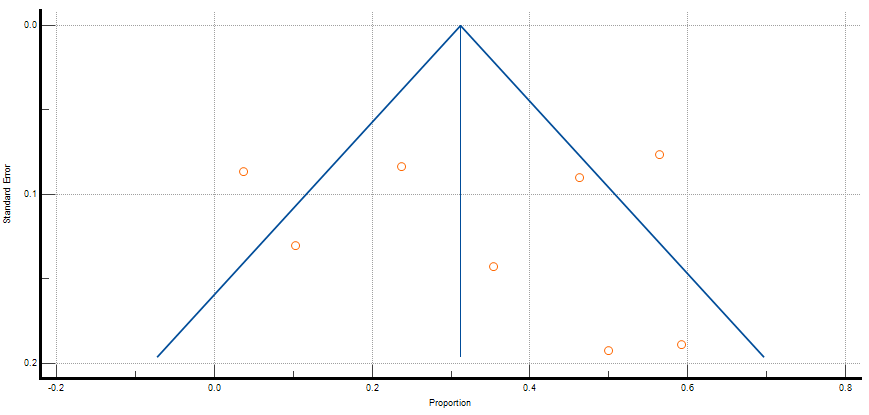


Levofloxacin


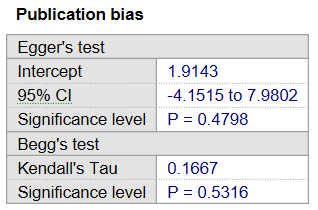


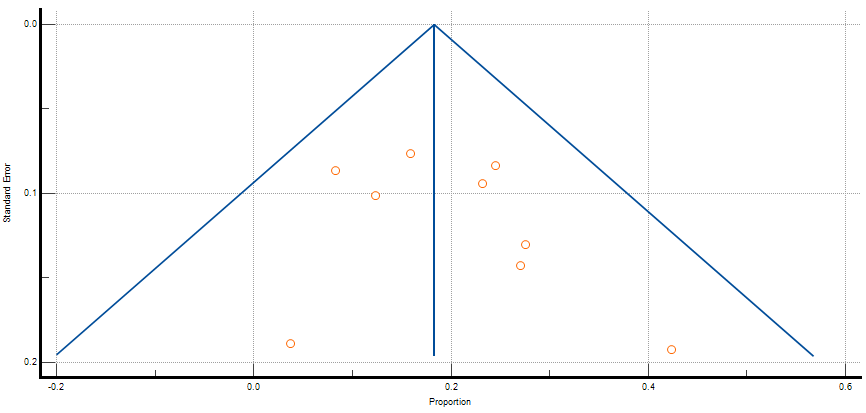


Tetracycline


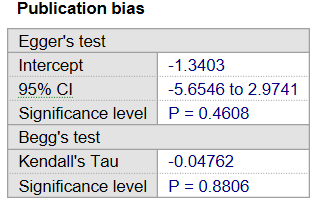


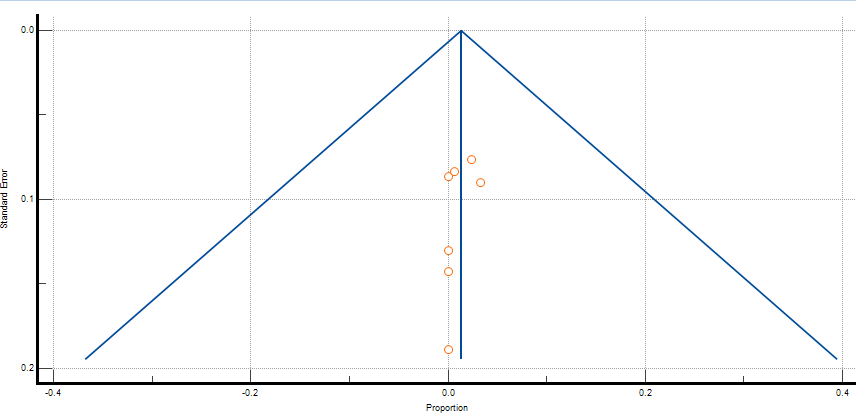


Rifampicin


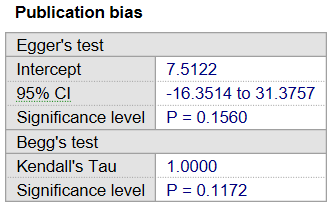


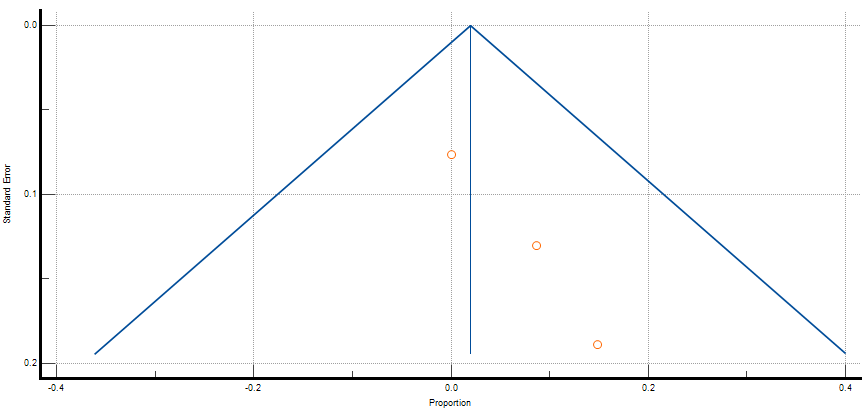


Dual therapy (metronidazole + clarithromycin)


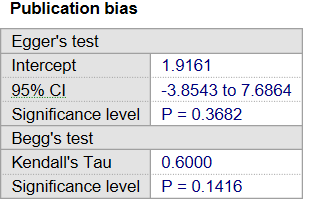


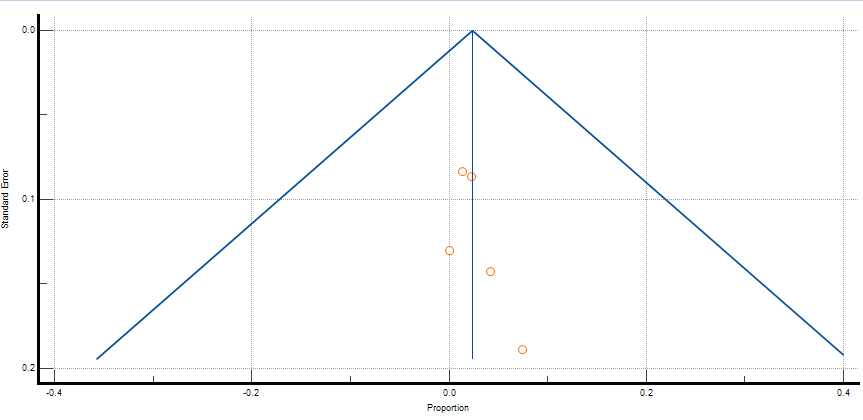

Supplement: Supplementary file 1 [file antibiotics-14-00524-s001.zip › File S2.doc]
